# Unsupervised water scene dehazing network using multiple scattering model

**DOI:** 10.1371/journal.pone.0253214

**Published:** 2021-06-28

**Authors:** Shunmin An, Xixia Huang, Linling Wang, Zhangjing Zheng, Le Wang

**Affiliations:** 1 Institute of Logistics Science and Engineering, Shanghai Maritime University, Shanghai, People’s Republic of China; 2 College of Ocean Science and Engineering, Shanghai Maritime University, Shanghai, People’s Republic of China; Vellore Institute of Technology: VIT University, INDIA

## Abstract

In water scenes, where hazy images are subject to multiple scattering and where ideal data sets are difficult to collect, many dehazing methods are not as effective as they could be. Therefore, an unsupervised water scene dehazing network using atmospheric multiple scattering model is proposed. Unlike previous image dehazing methods, our method uses the unsupervised neural network and the atmospheric multiple scattering model and solves the problem of difficult acquisition of ideal datasets and the effect of multiple scattering on the image. In our method, in order to embed the atmospheric multiple scattering model into the unsupervised dehazing network, the unsupervised dehazing network uses four branches to estimate the scene radiation layer, transmission map layer, blur kernel layer and atmospheric light layer, the hazy image is then synthesized from the four output layers, minimizing the input hazy image and the output hazy image, where the output scene radiation layer is the final dehazing image. In addition, we constructed unsupervised loss functions which applicable to image dehazing by prior knowledge, i.e., color attenuation energy loss and dark channel loss. The method has a wide range of applications, with haze being thick and variable in marine, river and lake scenes, the method can be used to assist ship vision for target detection or forward road recognition in hazy conditions. Through extensive experiments on synthetic and real-world images, the proposed method is able to recover the details, structure and texture of the water image better than five advanced dehazing methods.

## Introduction

Haze is a common atmospheric phenomenon that in water conditions such as oceans, rivers, lakes, etc. In these conditions, the frequent occurrence of haze often causes the captured images to lose detailed information, and affects lots of visual tasks in water conditions, such as target detection and object classification. So far, although many methods of image dehazing have been proposed and achieved desired effects [1–6], the dehazing algorithms for water conditions have not been paid much attention to. Unlike land conditions, there is a lot of dense haze in water conditions and it is difficult to collect ideal data sets. Some image dehazing methods suitable for land conditions will result in halo and blur when used in water conditions. There are two reasons for this phenomenon. On the one hand, the haze in water conditions is dense, and multiple scattering has a great influence on the image. But the image dehazing algorithms of land conditions only consider single scattering. So, the blur phenomenon will appear when use the atmospheric single scattering model to remove haze in water conditions. On the other hand, it is difficult to collect ideal datasets in water conditions, and the model trained with the datasets of land conditions will cause halo phenomenon when applied to water conditions. The main contributions of our method of image dehazing in water conditions are as follows: the unsupervised network and atmospheric multiple scattering model are adopted in this method, which solves the problem of the difficulty of collecting ideal datasets and the influence of multiple scattering on the image. In addition, this method has a wide range of application prospects. The changing environment in the real hazy condition will reduce the effect of dehazing of the pre-trained model. But the unsupervised method and multiple scattering model have been adopted in our method, which could be adapted to the changeable real-world haze conditions well, for example: in the ocean condition, the haze is thick and changeable, so this method can assist the visual analysis of shipping channel. In the following, we will introduce the existing image dehazing methods and our proposed method.

Physical models of imaging in hazy conditions can be divided into atmospheric single scattering model [7] and atmospheric multiple scattering model [8]. Among them, Wang et al. [8] and He et al. [9] used the atmospheric multiple scattering model for dehazing, which achieved better effect compared with the atmospheric single scattering model. According to the atmospheric scattering model proposed by Narasimhan and Nayar [7, 10], it can be seen that the atmospheric single scattering model is a degenerate form of the atmospheric multiple scattering model, and the atmospheric multiple scattering model takes into account the interference caused by multiple scattering to the image, while the atmospheric single scattering model only considers the case of single scattering. Therefore, multiple scattering is widespread in dense haze conditions, and the method of dehazing using atmospheric single scattering model often fails to achieve the desired effect. At present, image dehazing methods can be roughly divided into two categories: the prior-based method and the learning-based method. It should be pointed out that the two methods use the atmospheric single scattering model as the physical model to the most extent. The prior-based method manually extracts the features of the hazy image through observation and hypothesis [1, 3, 4, 11, 12]. For example, He et al. [11] proposed a method based on dark channel prior knowledge statistics to solve the problem of image dehazing, and got haze-free images by this prior. But almost all the methods of image dehazing that based on the prior rely too much on prior knowledge, which result in unsatisfactory dehazing effects in certain changing conditions. In order to reduce the dependence on prior knowledge, the learning-based method has attracted extensive attention. Unlike the prior-based method, the parameters of the atmospheric single scattering model are learned from the training data set in the learning-based method [2, 13–20]. For example, Cai et al. [14] proposed an end-to-end method of image dehazing for the problem of image dehazing that relies too much on prior knowledge. Cai et al. [14] automatically extracted the characteristics of haze in hazy images and successfully estimated the transmission map through neural network, and finally achieved dehazing results. But almost all learning-based methods of image dehazing are carried out through supervision and training. Therefore, if the gap between the training set and the real condition is too large, its effect is still not ideal. But the unsupervised image enhancement method only focuses on to the information of the input image itself and does not require a large number of training sets to train the network model, it has attracted widespread attention from researchers in recent years. At the same time, a variety of image enhancement methods based on unsupervised learning have been proposed [5, 21–23]. In summary, in the prior-based image dehazing methods, there are too much reliance on the prior knowledge, resulting in suboptimal dehazing in certain changing scenes. In learning-based image dehazing methods, ideal datasets are difficult to collect, so network models trained on synthetic datasets are not ideal for application to real-world scenes. Image dehazing has been little studied in unsupervised image enhancement methods, and the above methods do not consider the effect of multiple scattering on the image.

We noticed that the haze in the water condition is dense and it is difficult to collect he ideal dataset. Previous methods of image dehazing dehaze through the atmospheric single scattering model and synthetic dataset, so the dehazing effect is not ideal. Unlike previous dehazing methods, we proposed a reconstructed atmospheric multiple scattering model and an unsupervised dehazing network for dehazing in water condition, which solved the problem of the effect of multiple scattering on images and the difficulty of collecting ideal data. Specifically, the reconstructed atmospheric multiple scattering model simply views the convolution of the blur kernel and the haze-free image as Hadamard product. The unsupervised dehazing network uses four branches (J-Net, T-Net, K-Net, and K-estimation module) and decomposes the atmospheric multiple scattering model into four constituent layers, i.e., scene radiation layer, transmission map layer, blur kernel layer, and atmospheric light layer, which makes the atmospheric multiple scattering model be embedded in the unsupervised network well. It should be noted that there are three unsupervised branches and one prior-based branch in the unsupervised dehazing network. The three unsupervised branches and one prior-based branch are used to estimate the scene radiation layer, the transport map layer, the blur kernel layer and the atmospheric light layer respectively. In addition, we have constructed unsupervised losses based on prior knowledge, including color attenuation energy loss and dark channel loss, which are used to constrain sub-networks. Finally, the unsupervised dehazing network reconstructs the four layers of output into the hazy image in a self-supervised manner, and minimizes the reconstruction loss of the reconstructed hazy image and the input hazy image. In this process, the output scene radiation layer is the haze-free image.

To sum up, the main work of the proposed method is in three aspects:

- For the effect of multiple scattering on the image in the water condition, we have proposed to dehaze through atmospheric multiple scattering model in water condition. Specifically, the convolution of the blur kernel and the clean image is simply viewed as Hadamard product.
- For the problem that it is difficult to collect datasets in the water condition, we have proposed to dehaze through an unsupervised dehazing network for water conditions. Specifically, the unsupervised dehazing network consists of three unsupervised branches and one prior-based branch, which are used to estimate the scene radiation layer, the transmission map layer, the blur kernel layer and the atmospheric light layer. The whole network synthesizes hazy images in a self-supervised manner, and the final output scene radiation layer is a haze-free image. The unsupervised dehazing network can only focus on the features of the real-world image itself and avoid intensive operations on the dataset.
- In order to constrain the output of the branch of unsupervised network, we have proposed an unsupervised loss function. Specifically, the colour attenuation energy loss function and the dark channel loss are constructed based on the colour attenuation prior [12] and the dark channel prior respectively, which are used to constrain the J-Net and the T-Net.

The structure of this paper can be divided into the following four sections: The first section explains the problems about dehazing in water conditions, the advantages and disadvantages of existing methods of image dehazing and our contribution. In the second section, we provide an overview of the relevant work, mainly including the atmospheric scattering physical model, prior-based method, learning-based method and unsupervised image enhancement method. In the third section, we analyze and explain the proposed method from the reconstructed atmospheric multiple scattering model and the unsupervised dehazing network. It should be pointed out that in the unsupervised dehazing network, the realization methods of the four branches (J-Net, T-Net, K-Net and A-estimation module) are introduced respectively. In the fourth section, we demonstrate the advantages of the proposed method through qualitative and quantitative experiments.

## Related work

In this part, firstly, the physical model of atmospheric scattering was introduced, which includes the atmospheric single scattering model and the atmospheric multiple scattering model. Secondly, the prior-based dehazing methods and the learning-based dehazing methods were introduced respectively. Finally, the application of unsupervised neural network in image enhancement was introduced.

### Atmospheric scattering model

Atmospheric scattering physical models [10] can be divided into atmospheric single scattering model [7] and atmospheric multiple scattering model [8]. Among them, Wang et al. [8] and He et al. [9] carried out dehazing work through the atmospheric multiple scattering model and achieved better dehazing effects than using the atmospheric single scattering model. According to the atmospheric scattering model proposed by Narasimhan and Nayar [7, 10], the atmospheric single scattering model is a degenerate form of the atmospheric multiple scattering model, and the atmospheric multiple scattering model takes into account the interference caused by multiple scatterings to the image, while the atmospheric single scattering model only considers the case of single scattering.

#### Atmospheric single scattering model

**Fig 1:**
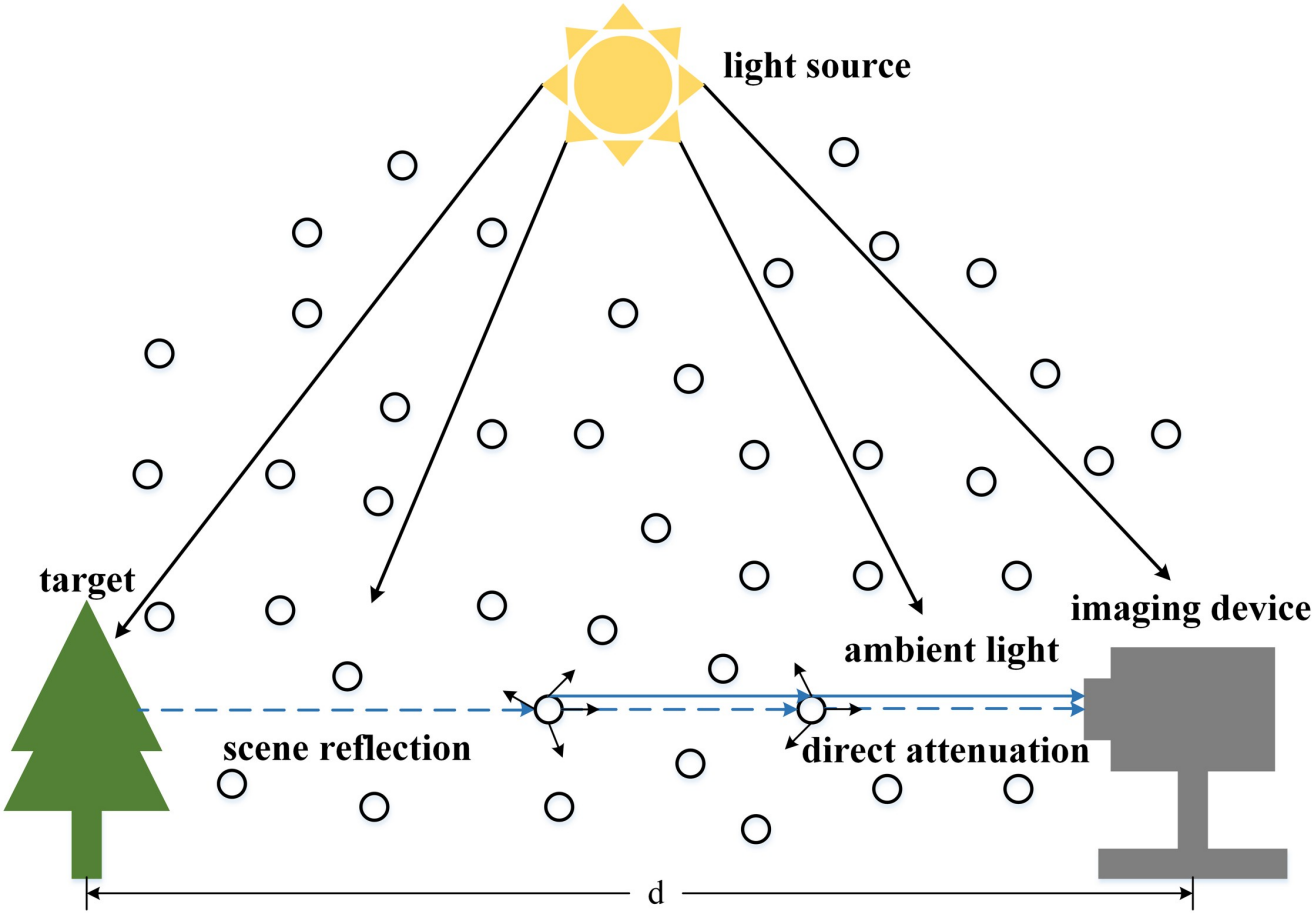
The imaging process using the atmospheric single scattering model, which ignores the multiple scattering of light in the atmosphere, but only considers the single scattering of light in the atmosphere.

Fig 1 shows the process of atmospheric single scattering, and the atmospheric single scattering model can be expressed as:

I(x)=t(x)∙J(x)+[1−t(x)]∙A. (1)

In this equation, the hazy image is represented by *I*(*x*); The haze-free image is represented by *J*(*x*); *A* represents the global atmospheric light, and *t*(*x*) is the transmission map, which can be expressed in the following ways:

$$t(x)=e^{-\beta d(x)},$$ (2)

here *β* is the scattering coefficient; *d*(*x*) is the distance between the target and the imaging device.

#### Atmospheric multiple scattering model

**Fig 2:**
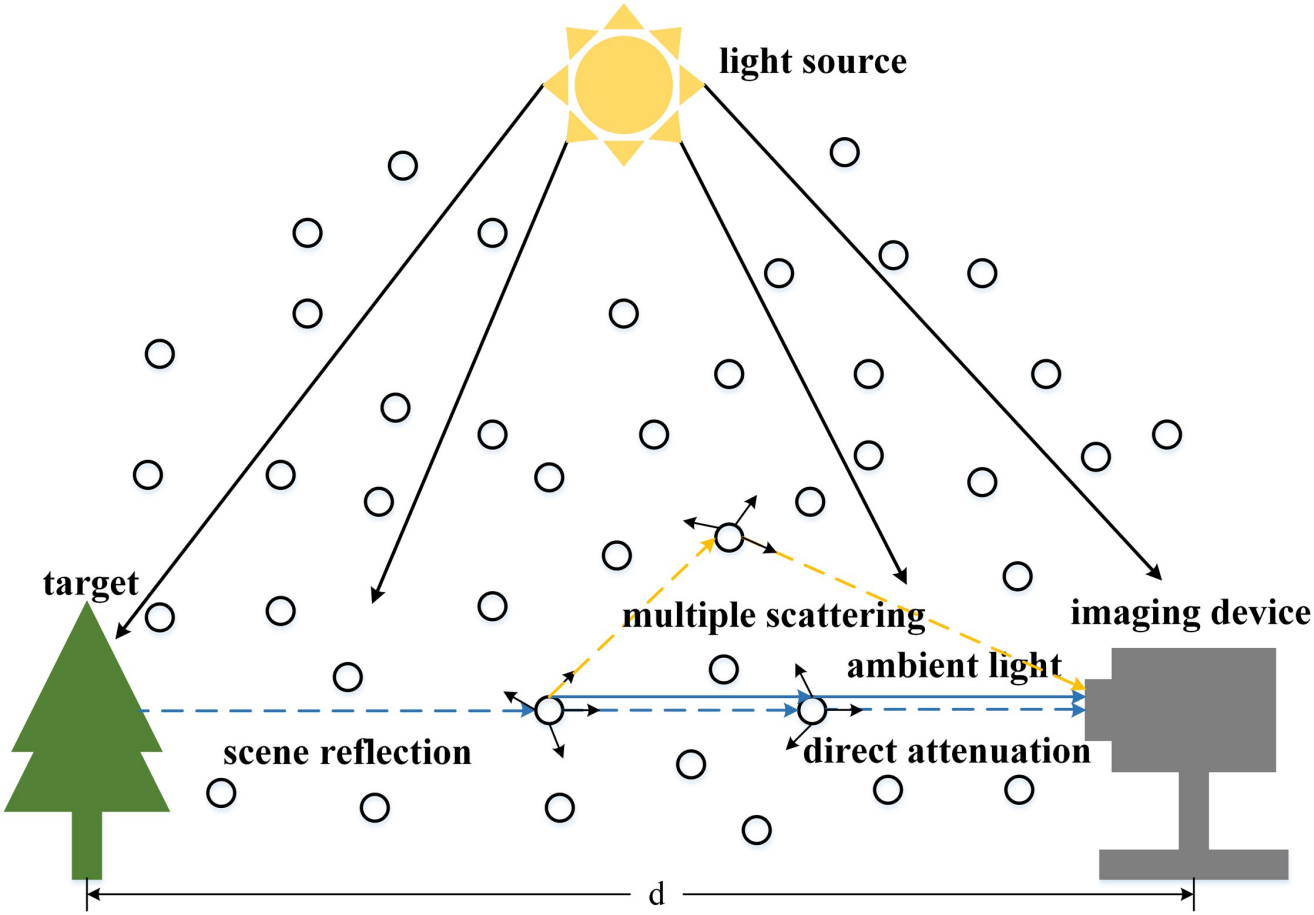
The imaging process using the atmospheric multiple scattering model. Different from the atmospheric single scattering model, the atmospheric multiple scattering model takes into account the multiple scattering of light in the atmosphere and can describe the interaction between the reflected light from different sites.

Fig 2 shows the process of atmospheric multiple scattering [8], and the atmospheric multiple scattering model can be expressed as:

$$I(x)=[J(x)*h_{A}]∙t(x)+[1-t(x)]∙A,$$ (3)

Here *h*_*A*_ is the convolution matrix, and it is the presence of the convolution matrix that causes the blurring of the image. Many researchers solve the convolution matrix through filtering or Monte Carlo methods [24–26], but these methods are too complicated. For simplicity of representation, we call the convolution matrix as the blur kernel and denote it by *k* in the following. Here * is the convolution operation. Under the influence of multiple scattering, different scene points produce aliasing on the imaging plane and cause the result of imaging to be blurred.

### The prior-based method

The prior-based dehazing method processes hazy images through the observation and statistics of hazy images and manually extracts the prior. Many prior-based methods have been proposed based on different observations and assumptions [1, 3, 4, 11, 12]. For example, He et al. [11] proposed a statistics method based on the prior knowledge of dark channel for image dehazing. This method thinks that there are some pixels in the local area of haze-free image, and at least one of the colour channels’ value is very small or even close to zero. Based on this observation, it performs dehazing processing on the input hazy image. Although the dark channel prior method can effectively solve the problem of image dehazing in outdoor conditions, the overall result of dehazing is dark and the sky area is prone to distortion. Zhu et al. [12] proposed a statistical method based on the prior knowledge of colour attenuation for the degradation of dehazing result. This method think that the depth of a haze-free image is positively correlated with the difference between its brightness and saturation. Based on this observation, this method models the depth of the scene and successfully restores haze-free image. In addition, non-local colour prior (NCP) [3] and haze-line prior (HLP) [4] have also been proposed based on observations and assumptions. Although these prior-based methods have achieved good results in many scenes, the priors through manually extracted may be inconsistent with the information of environmental, and the dehazing effect cannot always achieve the desired results.

### The learning-based method

Deep learning methods are currently used to solve many problems [27–30]. Unlike the prior-based method, the learning-based method adopts a data-driven approach and try to directly estimate the intermediate transmission map, the atmospheric light map and the final dehazing result. In order to avoid relying too much on prior information, many researchers completed the image dehazing task through convolutional neural network instead of manually extracting the prior. Therefore, some image dehazing methods through neural networks have been proposed [2, 13–20]. For example, Cai et al. [14] proposed an end-to-end image dehazing method for the problem of image dehazing that relies too much on prior knowledge. They automatically extract the features of haze in hazy images and successfully estimated the transmission map through neural network, and finally realized image dehazing. Although the convolutional neural network can automatically extract the characteristics of haze in the hazy image and successfully estimate the transmission map, the recovered dehazing results are prone to network artifacts because of block training during the training process. Aiming at the problem of estimation of atmospheric light, Zhang et al. [16] proposed to estimate the transmission map and atmospheric light simultaneously. This method can estimate the transmission map and atmospheric light at the same time by integrating the atmospheric scattering model into the neural network model through convolutional neural network and successfully recovered the haze-free image. Although the transmission map and atmospheric light can be estimated at the same time through the convolutional neural network, the recovered dehazing result still has a certain amount of haze, and the effect is general under dense haze. It should be pointed out that although learning-based image dehazing methods are widely used, their results of dehazing are sub-optimal because of using atmospheric single scattering model and data-driven model.

### Unsupervised image enhancement method

Since the above shortcomings of learning-based image enhancement methods, unsupervised image enhancement methods have been proposed [5, 21–23]. For instance, in response to the problems with data-driven learning methods, Ulyanov et al. [22] proposed a deep image prior method (DIP). This method can restore the input degraded picture to an undegraded picture by using an untrained neural network and an early stopping strategy. Although this method can achieve image enhancement, the parameters that can be estimated by the neural network are limited and it is difficult to determine the early stopping time, which will cause a direct impact on the final result. In response to the problem of prior parameter estimation of image depth, Irani et al. [23] proposed a new method of unsupervised image enhancement. They solved complex image enhancement problems by coupling multiple DIPs and using the idea of layer separation, such as image segmentation, watermark removal, etc. Although this method can solve complex image enhancement problems through multiple DIPs, it still uses the early stopping strategy when fits the network model, which will not be conducive to the estimation of the optimal result. Li et al. [5] proposed a new unsupervised image dehazing method for the problem with the early stopping strategy. They inputted the hazy image into the unsupervised network and constrained unsupervised network by minimizing the loss of input hazy image and output synthetized hazy image. Although this method can achieve unsupervised dehazing, the final output is still sub-optimal because of using the atmospheric single scattering model. This method is hereafter referred to as YOLY [5].

Although the proposed method and the YOLY both use unsupervised method for image dehazing, they are quite different. Firstly, in principle, YOLY is not a dehazing method specially designed for water conditions. The proposed method solves the dehazing problem in water conditions by using atmospheric multiple scattering model, while YOLY uses atmospheric single scattering model. But in the water condition, the dehazing result by using the atmospheric single scattering model is suboptimal. Secondly, according to the characteristics of hazy waters, the prior knowledge is fused with the neural network, while YOLY only uses the neural network to estimate the information of each layer. In addition, the structure of network and loss function are also different. In terms of the structure of network, the proposed method designed four branches to estimate the information of different layers according to the reconstructed atmospheric multiple scattering model, while YOLY only contains three branches. In terms of loss function, unsupervised loss function was proposed for clean image estimation network and transmission image estimation network in the proposed method, while YOLY proposed unsupervised loss function for clean image estimation network and atmospheric light estimation network.

## Proposed method

In this section, the unsupervised dehazing network for water conditions by using the multi-scattering model will be illustrated. Specifically, the reconstructed atmospheric multiple scattering model and unsupervised dehazing network will be introduced.

### The reconstructed atmospheric multiple scattering model

**Fig 3:**
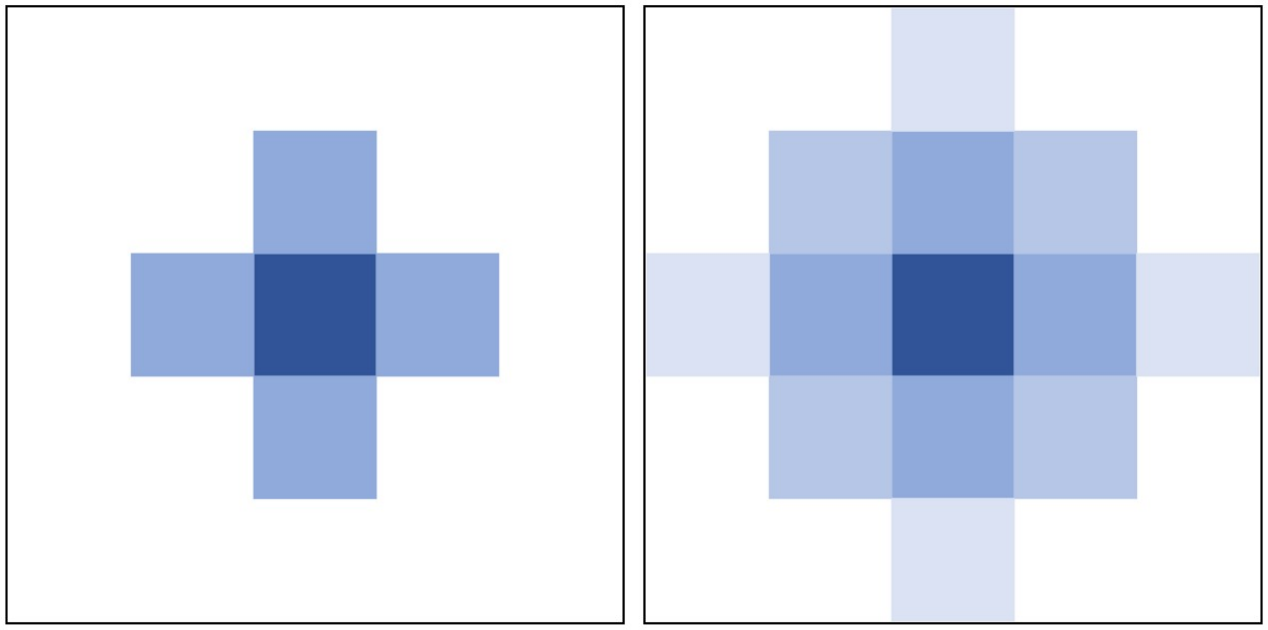
The dispersion spot generated by multiple scattering. Due to the influence of multiple scattering, the original clear image will be blurred, and the dispersion spot will be formed in local area.

In water conditions, many methods by using atmospheric single scattering model are prone to halo and blur because of multiple scattering in the dehazing results. According to the description in literature [8], because the reflected light enters the imaging device at different angles, it will form a dispersion spot caused by the multiple scattering and the clear image will become hazy. As shown in Fig 3, the image becomes blur caused by dispersion spot. Therefore, according to Eq (3) of atmospheric multiple scattering model, it can be deduced as follows:

$$J(x)*h_{A}=\frac{1}{t(x)}∙I(x)-A∙\frac{1}{t(x)}+A.$$ (4)

Therefore, it is a challenge to obtain blur kernel and make deconvolution operation. Many super-resolution methods have carried out targeted research on this problem, enlightenments can be obtained from some researches. For example, Gu et al. [31] proposed an image super-resolution method for the calculation of blur kernel and deconvolution. This method simply viewed the convolution of the feature map of the low-resolution image and the blur kernel as Hadamard product in the neural network, and finally restored the high-resolution image. In hazy images, blurring is widespread due to the influence of multiple scattering, and the halo and blur of the image are caused by the convolution of the blur kernel and the clean image. Therefore, according to Eq (4), the convolution of blur kernel and haze-free image can be simply viewed as a per pixel product. Therefore, the reconstructed atmospheric multiple scattering model can be expressed in the following way:

$$I(x)=[J(x)∙h_{A}]∙t(x)+A∙[1-t(x)].$$ (5)

### Unsupervised dehazing network

**Fig 4:**
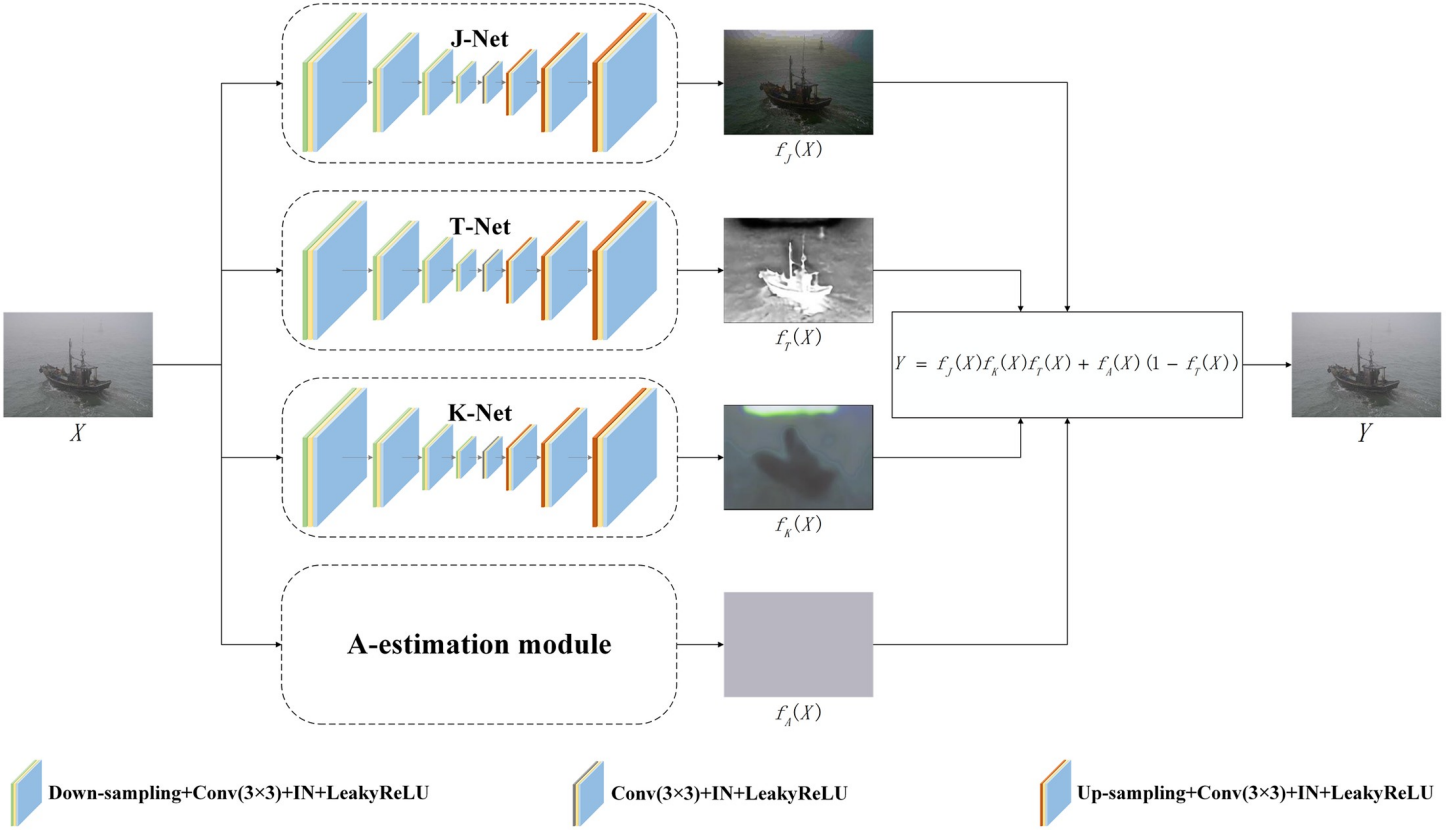
The proposed unsupervised dehazing network. In short, the unsupervised dehazing network consists of three unsupervised branches and a prior-based branch. The input hazy image is decomposed into four different layers by four branches, and then the four layers will be synthesized into hazy image according to the reconstructed atmospheric multiple scattering model.

In this section, a new unsupervised dehazing network will be introduced, the network structure is shown in Fig 4. The following sections will introduce the four branches of the unsupervised dehazing network and explain the constructed loss functions.

#### J-Net

**Fig 5:**
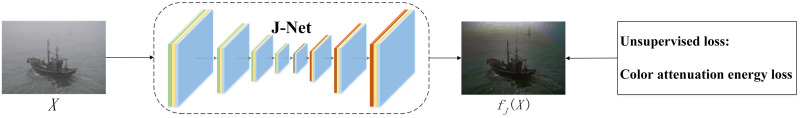
Clean image estimation branch, color attenuation energy loss is used for the constraint of network, the output of the network is a clean image with three-channel.

In the J-Net branch depicted in Fig 5, the U-shaped network [32] can increase the depth of the network and improve its learning ability. Specifically, J-Net consists of the convolution layer, the down-sampling layer, the up-sampling layer, the Instance Normalization layer [33] and the LeakyReLU activation function. In order to constrain the J-Net branch and generate haze-free image, the colour attenuation prior [12] is used to construct the unsupervised loss function, which is called the colour attenuation energy loss:

$$L_{J}={\left\|V(f_{J}(X))-S(f_{J}(X))\right\|}_{2}.$$ (6)

In this equation, the brightness and saturation of the haze-free image are represented by *V*(*f*_*J*_(*X*)) and *S*(*f*_*J*_(*X*)) respectively. According to the colour attenuation prior [12], the depth of the image is positively correlated with the difference between brightness and saturation in the haze-free image. Therefore, as the output of J-Net, the difference between the brightness value and saturation of *f*_*J*_(*X*) should be as close as possible. So that, J-Net can be constrained by L2 regularization of the difference between image’s brightness and saturation.

#### T-Net

**Fig 6:**
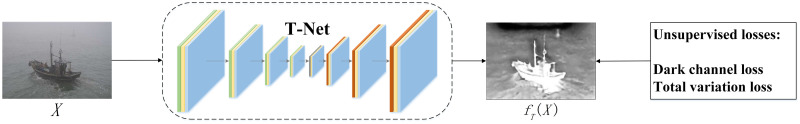
Transmission map estimation branch, dark channel loss and total variation loss are used to constrain the network whose output is a single channel transmission map.

In the T-Net branch depicted in Fig 6, the structure of T-Net is similar to the structure of J-Net. In order to constrain the generation of the transmission map of T-Net branch, the dark channel loss is constructed according to dark channel prior [11], and the total variation loss [34] is used to preserve the structure and details.

The dark channel loss has been incorporated the dark channel prior method. Specifically, first, the method of solving the transmission map through dark channel prior is used to solve the rough transmission map, and then do L2 regularization between the transmission map of the output of T-Net and the rough transmission map. Obtaining the dark channel of the haze-free image is the first condition. The dark channel can be expressed in the following way:

$$J^{dark}(x)=\underset{y\in \Omega (x)}{\text{min}}\left(\underset{c\in (r,g,b)}{\text{min}}\left(J^{c}(y)\right)\right).$$ (7)

According to the dark channel prior

$$J^{dark}(x)=0,$$ (8)

in the Eq (7), *x* and *y* are the pixel coordinates, *J*^*c*^ represents the c-th color channel of the haze-free image, Ω(*x*) is the local area centered on *x*, *J*^*dark*^ represents the dark channel of haze-free image. In addition, atmospheric light also needs to be required according to the dark channel. In the dark channel of the hazy image, the pixel with the largest pixel value in the top 0.1% of the image is selected to correspond to the hazy image, and the pixel with the highest pixel value is selected as atmospheric light. Then, according to the dark channel prior, after two minimum filtering, the equation can be obtained, equation “(see (9))”,

$$\underset{y\in \Omega (x)}{\text{min}}\left(\underset{c\in (r,g,b)}{\text{min}}\frac{I^{c}(y)}{A^{c}}\right)=\hat{t}(x)∙\;\underset{y\in \Omega (x)}{\text{min}}\left(\underset{c\in (r,g,b)}{\text{min}}\frac{J^{c}(y)}{A^{c}}\right)+1-\hat{t}(x),\;$$ (9)

where $\hat{t}(x)$ is the rough transmission map, and *A*^*c*^ is the atmospheric light solved by the dark channel prior. According to Eqs (7) and (8), the following equation can be obtained:

$$J^{dark}(x)=\underset{y\in \Omega (x)}{\text{min}}\left(\underset{c\in (r,g,b)}{\text{min}}\left(J^{c}(y)\right)\right)=0.$$ (10)

By substituting Eq (10) into Eq (9) for sorting, the rough transmission map can be expressed by the following equation:

$$\hat{t}(x)=1-\omega \underset{y\in \text{N}(x)}{\text{min}}\left(\underset{c\in (r,g,b)}{\text{min}}\frac{I^{c}(y)}{A^{c}}\right),$$ (11)

where *ω* is the correction factor, and the default is taken as *ω* = 0.95. According to the above process, the rough transmission map can be estimated by the method of dark channel prior [11], and then the rough transmission map and the transmission map predicted by T-Net can be L2-regularized to constrain the T-Net branch, which is called the dark channel loss. The dark channel loss function can be expressed in the following way:

$$L_{T}={\left\|f_{T}(X)-\hat{t}(x)\right\|}_{2},$$ (12)

the *f*_*T*_(*X*) represents transmission map predicted by T-Net, $\hat{t}(x)$ represents the rough transmission map estimated by dark channel prior [11]. In addition, in the total variation loss [34], L1 regularization is aim to constrain the gradient, which can preserve structure and details:

$$L_{t}={\left\|\nabla_{h}f_{T}(X)\right\|}_{1}+{\left\|\nabla_{v}f_{T}(X)\right\|}_{1},$$ (13)

where ∇_*h*_ and ∇_*v*_ represent the horizontal and vertical differential operation matrix respectively.

#### K-Net

In the K-Net branch, since the clean image, the transmission map and the blur kernel are subject to the input image, the similar network structures are used for J-Net, T-Net and K-Net. But the difference in K-Net is that it does not employ an explicit loss, but only guides optimization in a self-supervised manner.

#### A-estimation module

In the water condition, dense haze is a common phenomenon, and the changes of atmospheric light layer are relatively gentle. Therefore, the method of solving the atmospheric light layer through dark channel prior is used to solve the atmospheric light layer. That is, in the dark channel map, the pixel with the largest pixel value in the top 0.1% is selected to correspond to the hazy image, and the pixel with the highest pixel value is selected as atmospheric light layer.

After the four layers of output are obtained, the network needs to reconstruct the above four-layer output into a hazy image in a self-supervised manner and minimize the reconstruction loss function between the reconstructed hazy image and the input hazy image. The reconstruction loss function is composed of mean square error and structural similarity loss [35]. Therefore, the detailed information of the image and the structure and texture information of the image can be considered at the same time. The structural similarity loss can be expressed in the following way:

$$L_{S}=1-SSIM(X,Y),\;where$$

$$SSIM(X,Y)=\frac{\left(2\mu_{X}\mu_{Y}+C_{1})(2\sigma_{XY}+C_{2}\right)}{\left(\mu_{X}^{2}+\mu_{Y}^{2}+C_{1})(\sigma_{X}^{2}+\sigma_{Y}^{2}+C_{2}\right)},$$ (14)

where *X* and *Y* represent input hazy image and output hazy image respectively, *μ*_*X*_ and *μ*_*Y*_ represent the mean values of image *X* and *Y* respectively, *σ*_*X*_ and *σ*_*Y*_ represent the standard deviations of *X* and *Y* respectively, *σ*_*XY*_ represents the covariance of image *X* and *Y*, *C*_1_ and *C*_2_ are constants. The mean square error can be expressed as follows:

$$L_{MSE}={\left\|Y-X\right\|}_{2}.$$ (15)

Therefore, reconstruction loss can be expressed as follows:

$$L_{Rec}=\alpha L_{MSE}+\beta L_{S},$$ (16)

here *α* and *β* are the positive weights of each loss function.

In summary, the total loss of this model can be expressed as follows:

$$L=L_{Rec}+\gamma L_{J}+\delta L_{T}+\varepsilon L_{t},$$ (17)

here *γ*, *δ* and *ε* are the positive weights of each loss function.

## Experiments

In order to verify the effectiveness of the proposed method, comparative experiments have been carried out on the synthetic hazy image and the real hazy image, and the proposed method has been compared with the related dehazing method. The results show that the proposed method performs well on both synthetic and real images, especially in real water conditions, and the dehazing effect of the proposed method is superior to other dehazing methods. In the experiment, ten synthetic hazy images in water condition selected from the RESIDE data set were used for quantitative analysis, and ten real-world hazy images in water condition collected from the Internet were used for qualitative analysis. The synthetic image and the real image datasets can be found at: https://doi.org/10.17605/OSF.IO/Y46W5. The network was trained on a PC with Nvidia GeForce RTX 2060 GPU.

### Quantitative results on synthetic images

In the comparative experiment of synthetic images, ten synthetic hazy images in water condition selected from the RESIDE dataset were used for qualitative analysis, and the proposed method has been compared with five methods of dehazing. The five dehazing methods included priori-based dehazing methods and learning-based dehazing methods. The five dehazing methods are as follows: DCP [11], CAP [12], DehazeNet [14], AOD-Net [15], and GCAN [2]. To evaluate the comparison results from an objective perspective, PSNR [36] and SSIM [35] were set as objective evaluation metrics [37].

**Fig 7:**
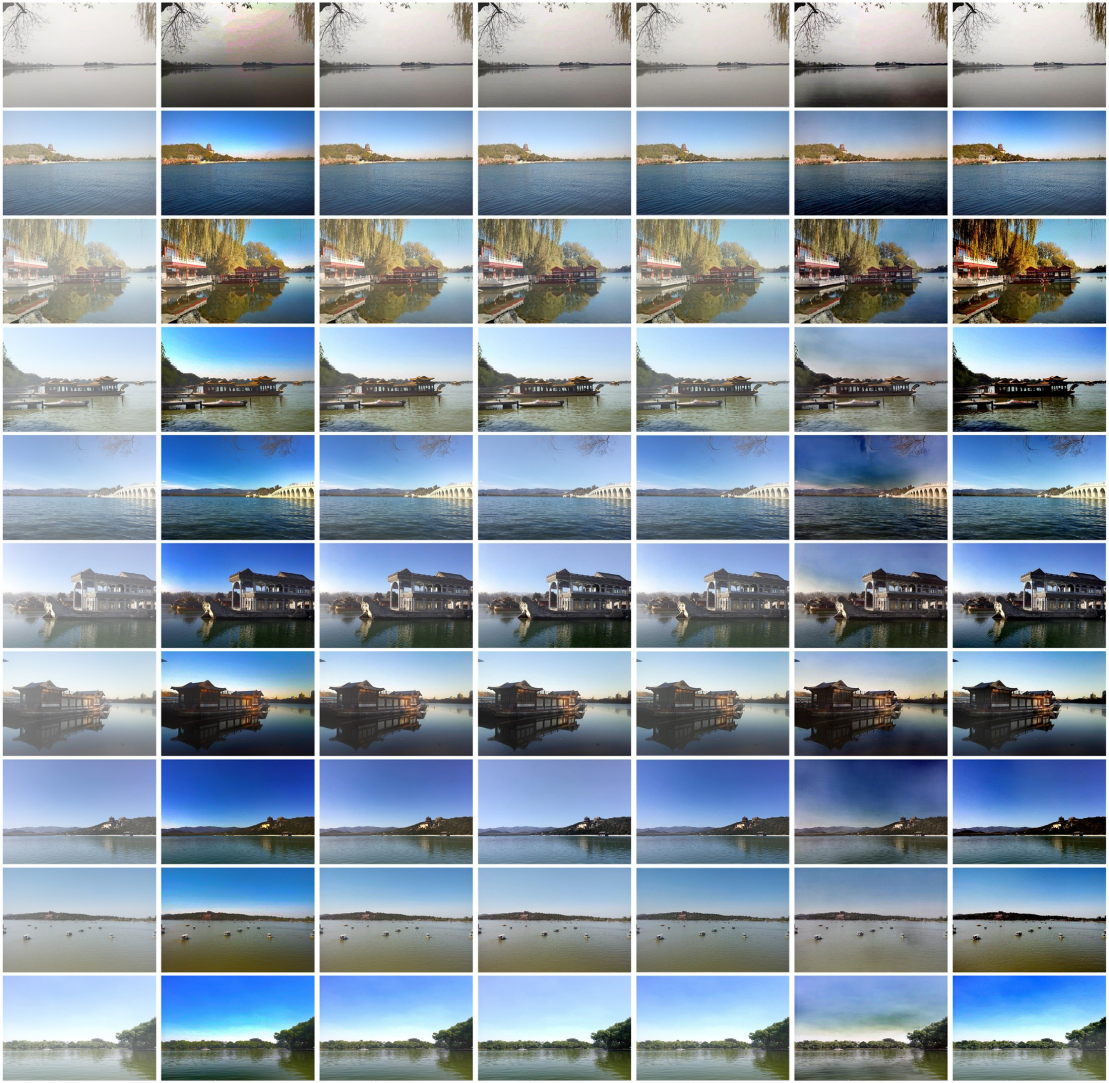
Qualitative comparison of different methods on synthetic images. From the left to the right column (i.e., Fig 7(a)–7(g)), the input hazy image, DCP [1], CAP [2], DehazNet [4], AOD-Net [5], GCAN [10], and our result).

According to Table 1, the proposed method is superior to the five dehazing methods in metric of PSNR and SSIM. Specifically, compared with the best prior-based method, the proposed method is 0.7 and 0.02 higher on PSNR and SSIM respectively. And compared with the best learning-based method, the proposed method is 1.19 and 0.01 higher on PSNR and SSIM respectively. This shows that the dehazing results recovered by the proposed method have a high degree of detail reduction and a complete structure. In addition, the best visual effect is shown as Fig 7, especially in the borders and detail areas of the images. For example, DCP [11] cannot restore the colour and texture information of the sky area well. For AOD-NET [15], it can be seen that there is still some haze in the image of dehazing results. However, the proposed method not only dehazes to the greatest extent, but also retains the colour and texture information. Therefore, the proposed method has excellent quantitatively and qualitatively results on the synthetic hazy image in water condition.

**Table 1:** The average results of SSIM and PSNR on the synthetic images.

Metrics	DCP	CAP	DehazeNet	AOD-Net	GCAN	Our result
PSNR	16.53	22.77	22.28	20.75	17.96	23.47
SSIM	0.91	0.93	0.92	0.93	0.94	0.95

### Qualitative visual results on real-world images

In order to show that the proposed method still has a good dehazing effect in the real-world scene, five dehazing methods also were used in comparison experiment with the real image. And the five dehazing methods are as follows: DCP [11], CAP [12], Dehazenet [14], AOD-Net [15], and GCAN [2].

**Fig 8:**
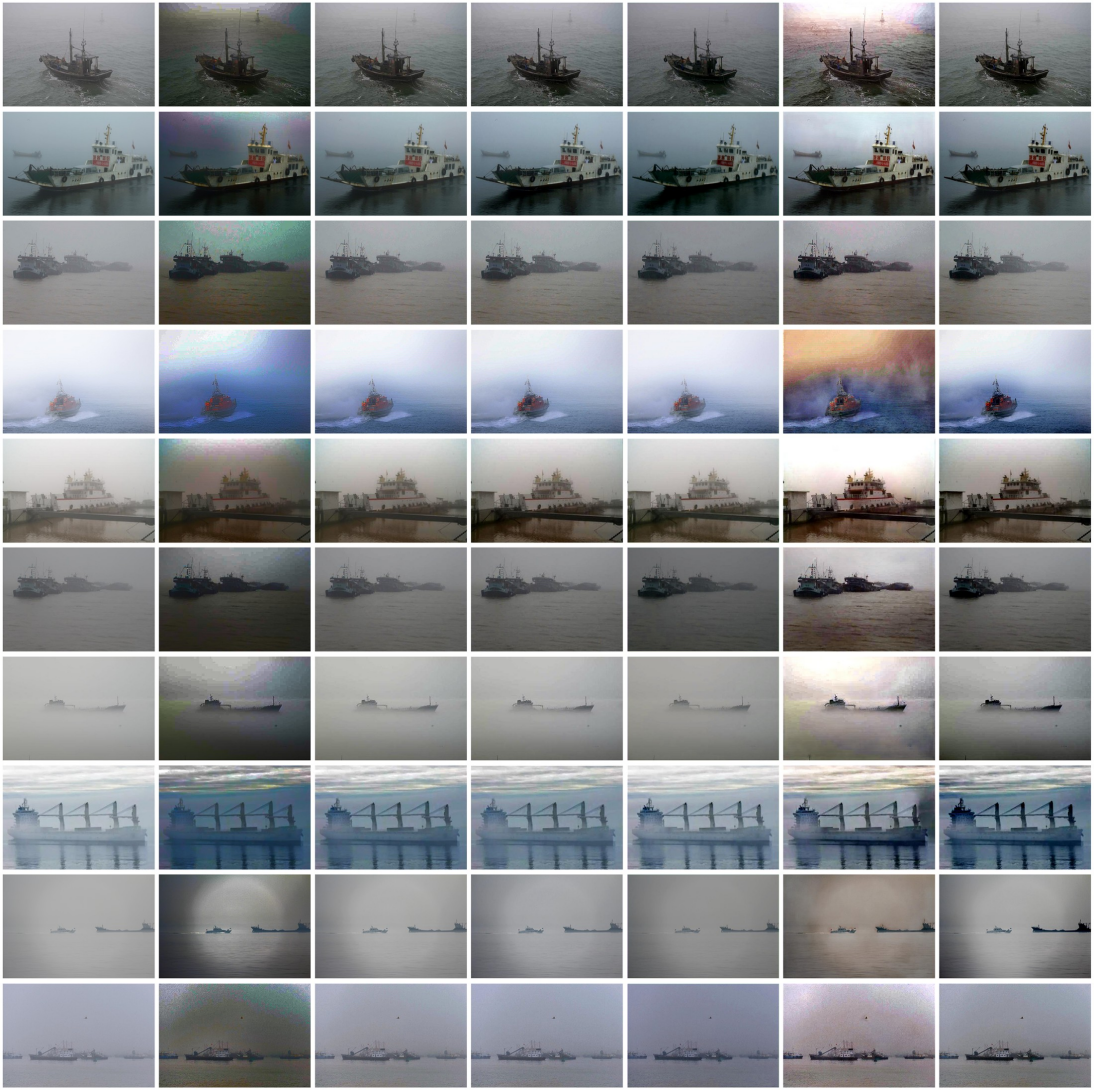
Quantitative comparison of different methods on real-world images. From the left to the right column (i.e., Fig 8(a)–8(g)), the input hazy image, DCP [1], CAP [2], DehazNet [4], AOD-Net [5], GCAN [10], and our result).

According to Fig 8, it can be seen that the DCP [11] results in a dark haze and color distortion in the sky area and at the edges of the image, which makes the visual effect of the dehazing result poor. For CAP [12], AOD-Net [15] and DehazeNet [14], their dehazing results are not only still unclear, but also blurred, and the dehazing effect is not obvious in the region with far depth of field. For GCAN [2], its dehazing result is brighter in visual effect, which compensates the information in the depth of field of the dehazing result. But its dehazing result has serious colour distortion and a layer of halo at the edge of the image. For the proposed method, it not only restores the colour of original image well, but also has a more thorough dehazing result. On the whole, the colour of the dehazing result is more realistic, without overexposure and distortion. In terms of details, the result of dehazing can reflect the details and edge information of the image well, without blurring and halo. Therefore, the proposed unsupervised dehazing network has a good dehazing effect on synthetic hazy image in water and real hazy image in water, and has excellent results in both quantitative and qualitative aspects.

## Conclusion

In this paper, an unsupervised dehazing network for water condition by using multiple scattering model was proposed. Compared with previous dehazing methods, the proposed network has three advantages: 1) Atmospheric multiple scattering model is used to dehaze in water conditions, which can effectively avoid the influence of multiple scattering on the image. 2) The unsupervised neural network is used for image dehazing. It divides the hazy image into scene radiation layer, transmission layer, blur kernel layer and atmospheric light layer, and does not use any information other than the content of input image. 3) In the unsupervised dehazing network, prior knowledge is fused into the neural network. Specifically, an unsupervised loss function incorporating prior knowledge is constructed in the network. And according to the characteristics of the water condition, the atmospheric light layer can be obtained by using prior knowledge. Compared with the learning-based methods, the proposed method does not require intensive operations on the data set. In addition, extensive experiments were conducted on synthetic images and real images, and the proposed method was compared with five state-of-the-art dehazing methods. Through quantitative and qualitative analysis, it is concluded that the proposed method has better dehazing effect than previous dehazing methods. In subsequent work, we will attempt to combine atmospheric multiple scattering model with semi-supervised learning to process real-world hazy images while ensuring real-time performance.

## Supporting information

S1 File Hazy images in water area. (ZIP)
