# Supplementary figures and images for: Unsupervised water scene dehazing network using multiple scattering model
Source: PLoS One. 2021 Jun 28;16(6):e0253214. doi: 10.1371/journal.pone.0253214 (PMC8238221; doi:10.1371/journal.pone.0253214)

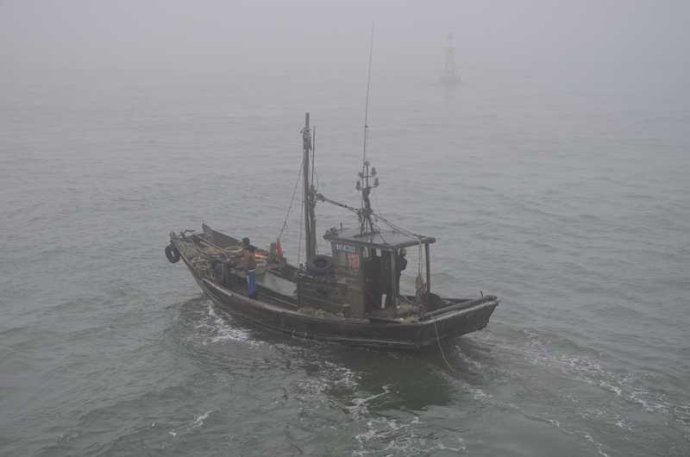

Supplement: S1 File — (ZIP) [file pone.0253214.s001.zip › Hazy images in water area/Real-world images/1.jpg]

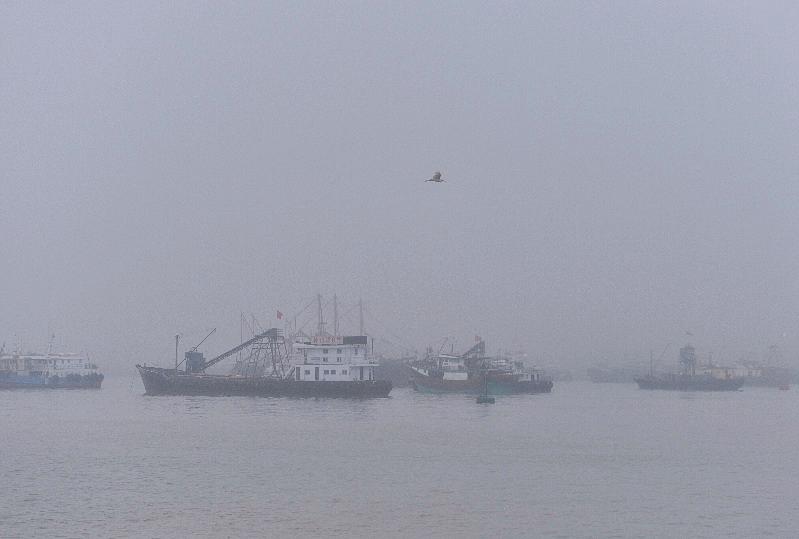

Supplement: S1 File — (ZIP) [file pone.0253214.s001.zip › Hazy images in water area/Real-world images/10.jpg]

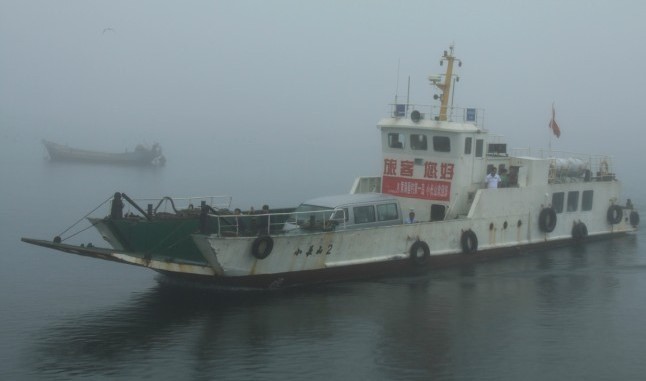

Supplement: S1 File — (ZIP) [file pone.0253214.s001.zip › Hazy images in water area/Real-world images/2.jpg]

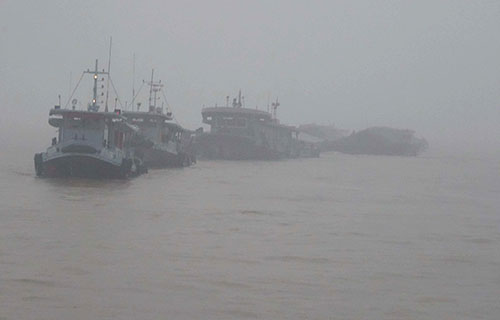

Supplement: S1 File — (ZIP) [file pone.0253214.s001.zip › Hazy images in water area/Real-world images/3.jpg]

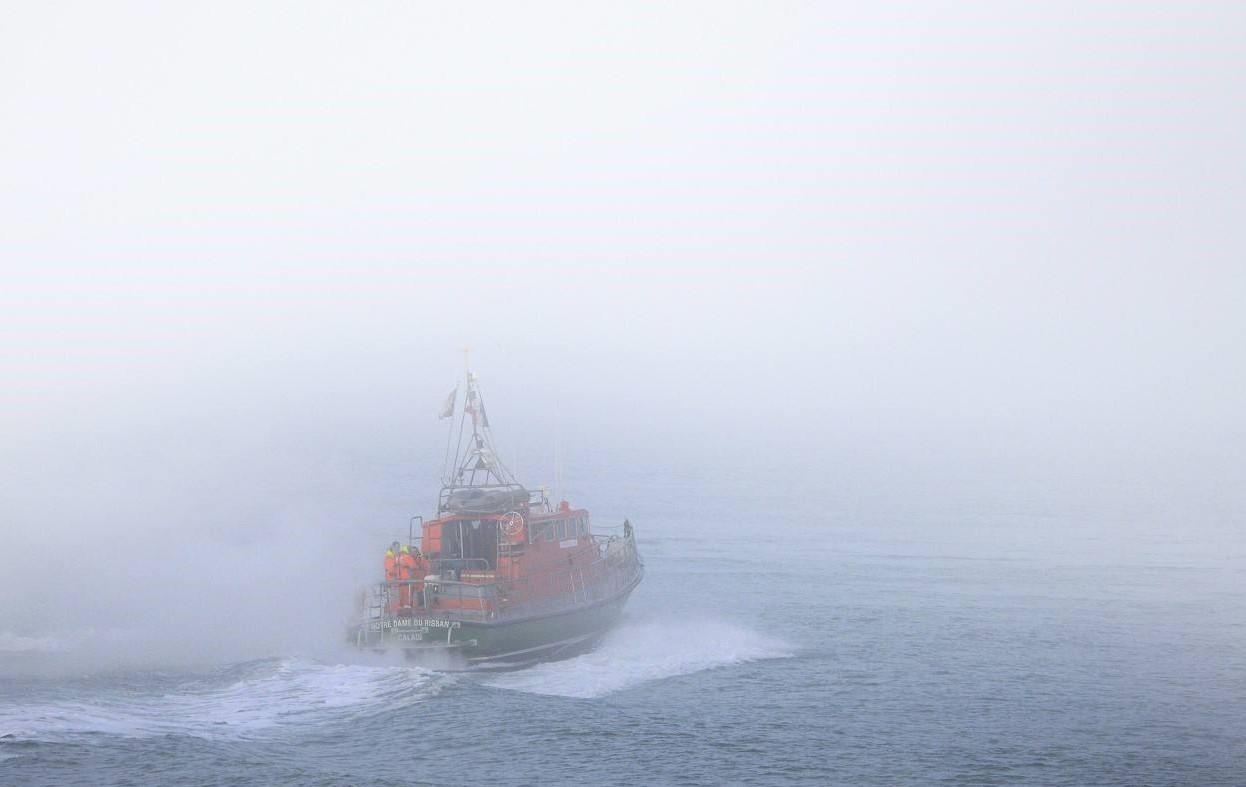

Supplement: S1 File — (ZIP) [file pone.0253214.s001.zip › Hazy images in water area/Real-world images/4.jpg]

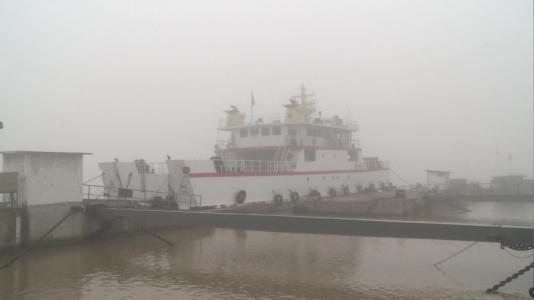

Supplement: S1 File — (ZIP) [file pone.0253214.s001.zip › Hazy images in water area/Real-world images/5.jpg]

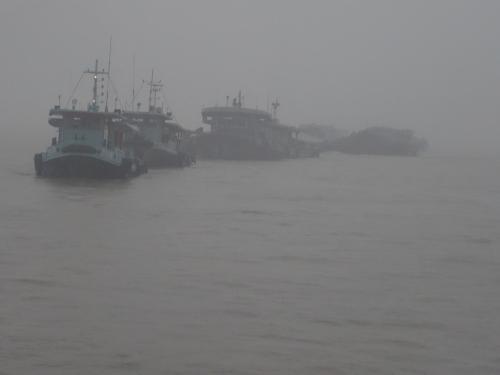

Supplement: S1 File — (ZIP) [file pone.0253214.s001.zip › Hazy images in water area/Real-world images/6.jpg]

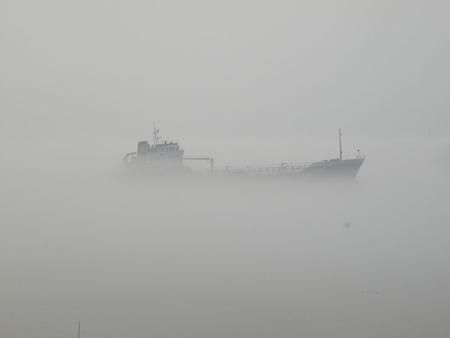

Supplement: S1 File — (ZIP) [file pone.0253214.s001.zip › Hazy images in water area/Real-world images/7.jpg]

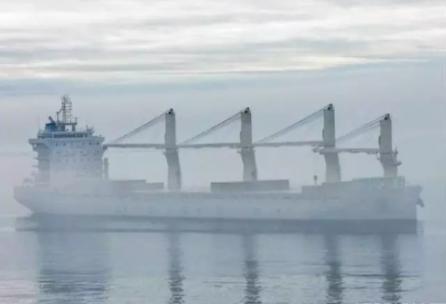

Supplement: S1 File — (ZIP) [file pone.0253214.s001.zip › Hazy images in water area/Real-world images/8.jpg]

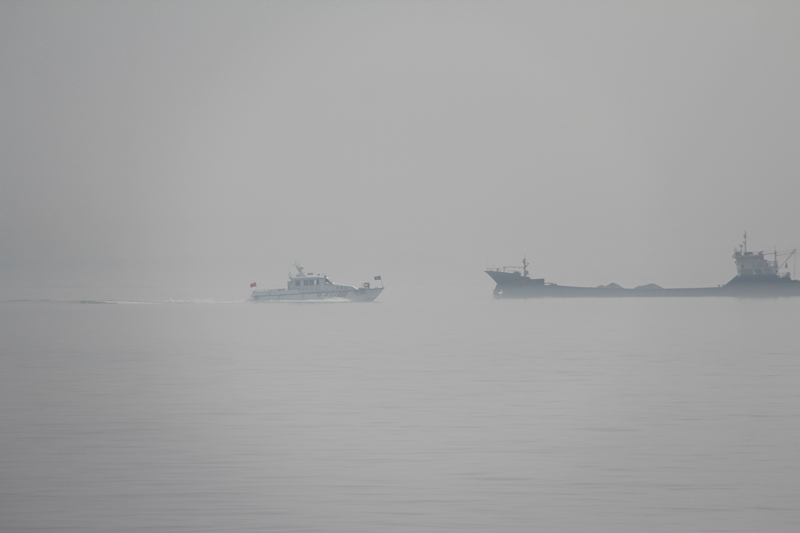

Supplement: S1 File — (ZIP) [file pone.0253214.s001.zip › Hazy images in water area/Real-world images/9.jpg]

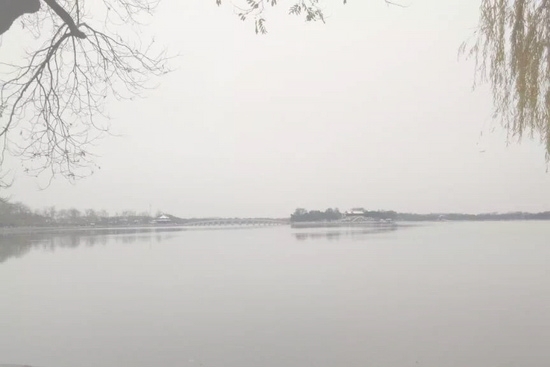

Supplement: S1 File — (ZIP) [file pone.0253214.s001.zip › Hazy images in water area/Synthetic images/1.jpg]

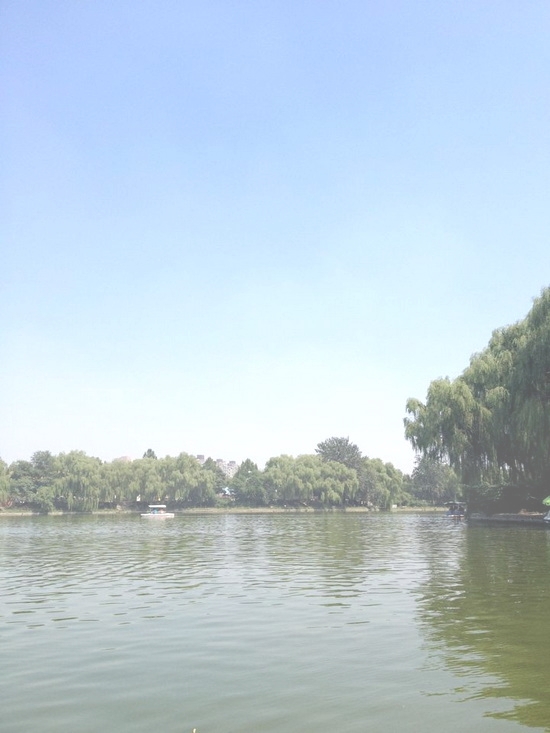

Supplement: S1 File — (ZIP) [file pone.0253214.s001.zip › Hazy images in water area/Synthetic images/10.jpg]

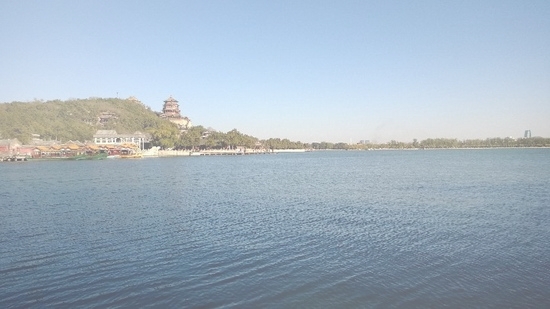

Supplement: S1 File — (ZIP) [file pone.0253214.s001.zip › Hazy images in water area/Synthetic images/2.jpg]

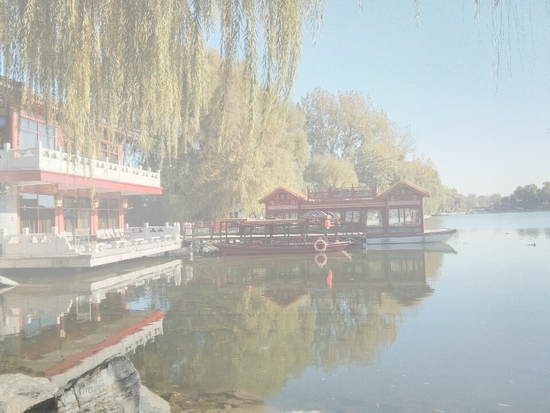

Supplement: S1 File — (ZIP) [file pone.0253214.s001.zip › Hazy images in water area/Synthetic images/3.jpg]

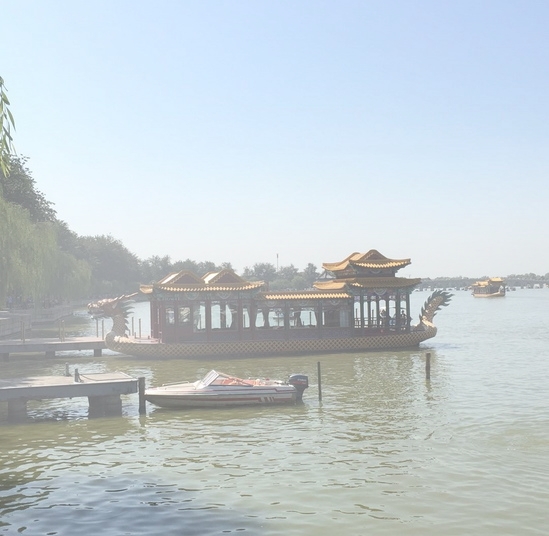

Supplement: S1 File — (ZIP) [file pone.0253214.s001.zip › Hazy images in water area/Synthetic images/4.jpg]

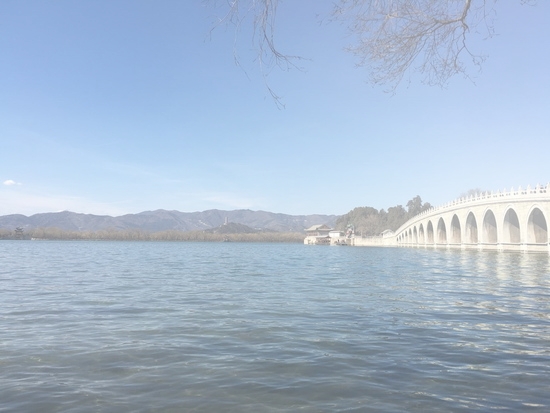

Supplement: S1 File — (ZIP) [file pone.0253214.s001.zip › Hazy images in water area/Synthetic images/5.jpg]

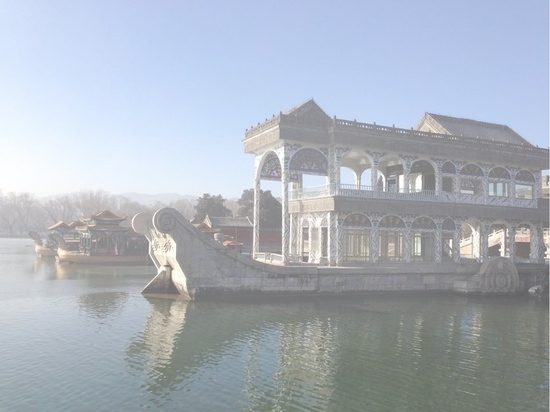

Supplement: S1 File — (ZIP) [file pone.0253214.s001.zip › Hazy images in water area/Synthetic images/6.jpg]

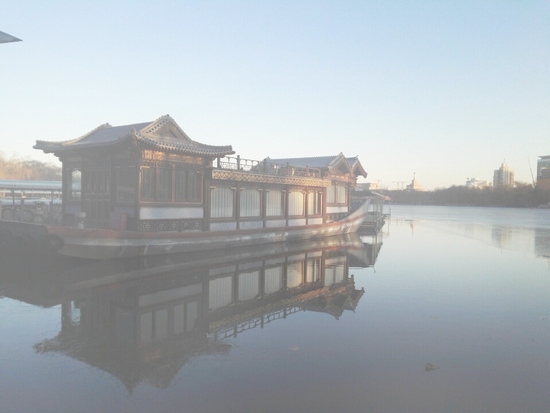

Supplement: S1 File — (ZIP) [file pone.0253214.s001.zip › Hazy images in water area/Synthetic images/7.jpg]

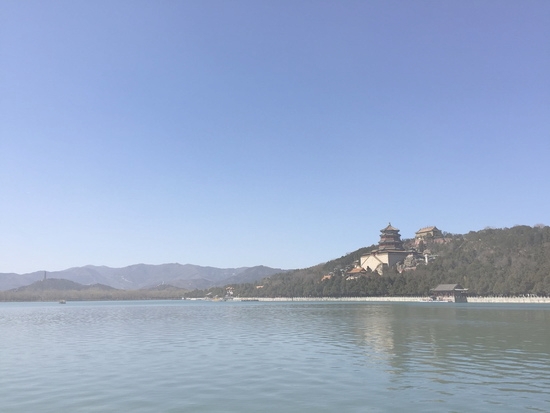

Supplement: S1 File — (ZIP) [file pone.0253214.s001.zip › Hazy images in water area/Synthetic images/8.jpg]

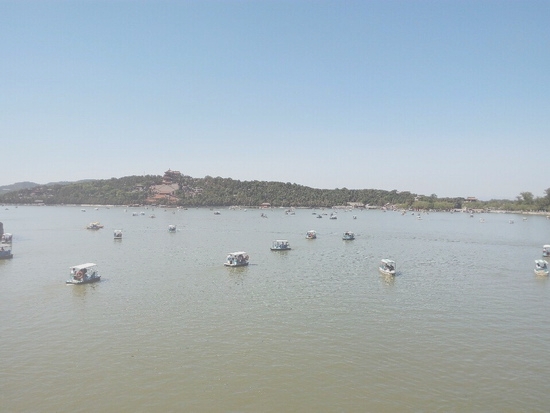

Supplement: S1 File — (ZIP) [file pone.0253214.s001.zip › Hazy images in water area/Synthetic images/9.jpg]
